# [Retracted] MicroRNA‑592 targets IGF‑1R to suppress cellular proliferation, migration and invasion in hepatocellular carcinoma

**DOI:** 10.3892/ol.2025.14941

**Published:** 2025-02-24

**Authors:** Wenyao Wang, Hongfei Zhang, Mao Tang, Longlong Liu, Zhengfang Zhou, Shaojun Zhang, Lichao Wang

Oncol Lett 13: 3522–3528, 2017; DOI: 10.3892/ol.2017.5902

Following the publication of this article, a concerned reader drew to the attention of the Editorial Office that the data panels for the Transwell cell invasion and migration assay data shown in Fig. 4A and B on p. 3526 contained a number of overlapping sections, such that these data apparently originated from the same original sources where the results from differently performed experiments were intended to have been portrayed. Moreover, certain of these data had already been submitted for publication in the following paper, written by different authors at different research institutes [Ni J, Yang Y, Liu D, Sun H, Jin S and Li J: MicroRNA-429 inhibits gastric cancer migration and invasion through the downregulation of specificity protein 1. Oncol Lett 13: 3845-3849, 2017].

Owing to the large number of overlapping data panels identified in Fig. 4A and B, and the fact that these data had already been submitted for publication in a different article to *Oncology Letters*, the Editor has decided that this paper should be retracted from the Journal. The authors were asked for an explanation to account for these concerns, but the Editorial Office did not receive a reply. The Editor apologizes to the readership for any inconvenience caused.

